# The impact of 24-h shift on cardiac anesthesia fellows

**DOI:** 10.3389/fpubh.2026.1807234

**Published:** 2026-06-10

**Authors:** Rongli Liu, Ken Shao, Lixian He, Yuntai Yao

**Affiliations:** 1 Jingmen Central Hospital, Jingmen Central Hospital Affiliated to Jingchu University of Technology, Jingmen, China; 2 Department of Anesthesiology, Fuwai Yunnan Hospital, Chinese Academy of Medical Sciences, Affiliated Cardiovascular Hospital of Kunming Medical University, Kunming, China; 3 Yunnan Provincial Cardiovascular Clinical Medical Center, Kunming, China; 4 Yunnan Provincial Cardiovascular Clinical Medical Research Center, Kunming, China; 5 Department of Anesthesiology, Fuwai Hospital, National Center for Cardiovascular Diseases, Peking Union Medical College and Chinese Academy of Medical Sciences, Beijing, China; 6 Center for Outcomes Research, Department of Anesthesiology, Critical Care and Pain Medicine, University of Texas, Houston, TX, United States; 7 Outcomes Research Consortium, Houston, TX, United States

**Keywords:** anesthesia, anesthesiologist, fellow doctors, on-call shift, questionnaire, sleep quality

## Abstract

**Objective:**

To investigate the workload, sleep quality, and psychological wellbeing of anesthesiologists at a large volume cardiovascular center.

**Methods:**

Eighteen anesthesiologists were enrolled from Fuwai Hospital. Data was collected using questionnaires and wearable smart wristbands. Participants worked 24-h shifts (from 8:00 a.m. to 8:00 a.m. the following day), during which they were responsible for emergency endotracheal intubation, invasive arterial catheterization, and central venipuncture catheterization, and analgesia pump management. Sleep parameters, physical activity, calories expenditure were recorded continuously throughout the shift using a wristband. The following self-report instruments were administered before and immediately after the shift: a self sleep quality rating, the Big Five Personality Traits, the Profile of Mood States (POMS), and the Karolinska Sleepiness Scale (KSS).

**Results:**

Fatigue scores on the POMS increased significantly after the 24-h shift (*P* < 0.05), whereas no other mood subscales showed statistically significant changes. Wristband data revealed limited sleep during the monitored duty period. KSS scores increased significantly from pre-shift to post-shift. The workload and physical monitoring data suggested that anesthesiologists experienced substantial physical and emotional strain during 24-h shifts.

**Conclusions:**

During a 24-h on-call shift, anesthesiologists obtained limited sleep and reported significantly increased fatigue and sleepiness post-shift. These findings emphasize the need for systemic changes in clinical scheduling to improve physician health and patient safety.

## Introduction

Anesthesiologists in China are experiencing severe work overload, accompanied by a concerning increase in sudden deaths. Between 1966 and 2015, 29 Chinese physicians reportedly died from cardiac arrest attributed to excessive workload, 14 of whom were anesthesiologists, with an average age of 35 years (1). In the first half of 2017 alone, six young anesthesiologists died from fatigue-related causes.

Anesthesiology is widely recognized as one of the most stressful medical specialties, and optimizing work schedules has been proposed as a strategy to mitigate physician burnout (2). Despite China's rapid economic growth over the past 30 years, disparities in the distribution of medical resources persist. The health care system has undergone major reforms to serve 19% of the global population; however, uneven distribution of human resources continues to create significant work-related stress. Pediatricians, emergency physicians, and anesthesiologists are in particularly short supply (3).

According to the National Health and Family Planning Commission of the People's Republic of China, the number of inpatient surgeries has doubled in the past decade, whereas growth in the anesthesiologist workforce has lagged far behind. Based on American and European standards (2.4 anesthesia providers per 10,000 people) (4, 5), China would require approximately 300,000 anesthesia providers. Currently, there are only 53,000 anesthesiologists and anesthesia residents, 6,700 assistants, and a negligible number of non-physician anesthesia providers (6). Consequently, overtime has become widespread.

Fatigue and handover-related risks have also been recognized as critical threats to patient safety. Against this backdrop, the present study investigates workload, sleep quality, and psychological status among anesthesia fellows in a large cardiovascular center during 24-h on-call duty.

## Methods

### Study design and participants

This observational study recruited 18 anesthesia fellows undergoing clinical training at a large cardiovascular center. The objective was to evaluate sleep patterns, physical activity, and psychological wellbeing before and after a 24-h on-call duty period. Because the study was conducted at a single institution and involved a limited number of eligible fellows during the study period, the final sample size was constrained by participant availability.

### Data collection

The study consisted of two main components:

1. **Self-reported questionnaires**. Participants completed structured questionnaires that collected demographic information, subjective sleep-related information, personality traits, mood states, and sleepiness. Demographic variables included sex, age, marital status, education, work experience, and professional title. Subjective sleep quality during the monitored 24-h on-call duty period was rated as “good”, “average”, “poor”, or “very poor”. Additional sleep-related questions included the presence of dreams (yes/no), number of dreams, dream valence (pleasant, unpleasant, or neutral), whether the dreams were work-related, and estimated sleep duration. Personality traits were assessed using the Big Five Personality Traits questionnaire. Mood states were assessed using the Profile of Mood States (POMS) (7). Subjective sleepiness was assessed using the Karolinska Sleepiness Scale (KSS) (8).

2. **Wearable physiological monitoring**. Each participant wore a smart wristband (Model V19) that continuously recorded estimated sleep-related parameters, including deep sleep, light sleep, rapid eye movement (REM) sleep, and total sleep duration, as well as activity-related parameters including step count, walking distance, and energy expenditure. Data were recorded for 24 h beginning at 8:00 a.m. on the first day of the on-call shift. Because this was a consumer wearable device rather than a research-grade actigraphy system, its sleep-stage estimates were interpreted cautiously.

### Psychological assessment instruments

The Big Five questionnaire evaluates five broad personality domains: extraversion, neuroticism, openness, agreeableness, and conscientiousness. Extraversion reflects sociability, activity, and positive emotionality. Neuroticism reflects emotional instability and proneness to anxiety, insecurity, and vulnerability. Openness reflects imagination, curiosity, and preference for novelty and new experiences. Agreeableness reflects trust, altruism, cooperation, and empathy. Conscientiousness reflects orderliness, self-discipline, responsibility, and goal-directed behavior.

The POMS is a validated 40-item instrument that evaluates transient affective states across six domains: tension-anxiety, depression-dejection, anger-hostility, vigor-activity, fatigue-inertia, and confusion-bewilderment. A Total Mood Disturbance (TMD) score is calculated by summing the negative mood domains and subtracting vigor, providing an overall estimate of affective state (7).

The KSS is a subjective measure of alertness rated on a 9-point scale: 1 = extremely alert, 2 = very alert, 3 = alert, 4 = rather alert, 5 = neither alert nor sleepy, 6 = some signs of sleepiness, 7 = sleepy but no effort to keep awake, 8 = sleepy with effort to stay awake, and 9 = very sleepy and fighting sleep (8).

### Study protocol

All questionnaires and wristbands were distributed at 8:00 a.m. on the first day of the on-call shift and collected 24 h later. Participants completed the questionnaire assessments at two time points: before shift (baseline, 8:00 a.m.) and after shift (after 24 h, at the completion of the on-call duty period). Each survey session required approximately 10–15 min to complete.

### Statistical analysis

Demographic and continuous variables are reported as mean ± SD or mean (range). Comparisons between before-shift and after-shift measurements were analyzed using paired two-tailed *t*-tests. Statistical significance was defined as a two-sided *P*-value < 0.05. Sample size considerations for this analysis were based on the power of a paired-samples two-tailed *t*-test for comparing pre- and post-shift measures of the Big Five scales. However, no formal *a priori* power calculation was performed, and the final sample size was constrained by participant availability during the study period. Given the exploratory nature of the study and the number of outcomes examined, the findings, particularly for secondary analyses, should be interpreted cautiously. All analyses were performed using SPSS software.

## Results

### Participant characteristics

Participants ranged in age from 28 to 40 years, with a mean age of 36 (Table 1). Thirteen were male (72%) and five female (28%). All were married. Educational background included Bachelor's degrees (33%), Master's (61%), and PhD (6%). Most had 5–10 years of experience (61%), while 39% had over 10 years. Thirteen were attending physicians, three were associate chiefs, and two were residents. Seven participants reported smoking or drinking; 11 did not. All participants reported no medication use for sleep.

**Table 1:** Participant demographics and professional characteristics.

Variable (*n* =18)	Mean ±standard deviation or *n* (%)
Age (years, mean and range)	36 ± 2.12
Male (*n*, %)	13 (72%)
Height (cm, mean and range)	168 ± 7.86
Weight (kg, mean and range)	64 ± 10.07
Marital status (*n*, %)	
Married	18 (100%)
Education level	
Undergraduate	6 (33%)
Master	11 (61%)
Doctor	1 (6%)
Work experience (*n*, %)	
5–10 years	11 (61%)
>10 years	7 (39%)
Professional title appraisal (*n*, %)	
Resident doctor	2 (11%)
Attending doctor	13 (72%)
Associate chief physician	3 (17%)
Alcohol taker and current smoker (*n*, %)	7 (39%)
Sleep–related medication user (*n*, %)	0 (0%)

### Sleep quality

As shown in Table 2, 61% of participants rated post-shift sleep quality as “average”, 28% as “good,” and 11% as “poor.” Dreams were reported by 44%, of which 33% were work-related. Average self-reported sleep duration was 6.25 ± 1.77 h.

**Table 2:** Subjective sleep quality and dream characteristics.

Item description	*n* (%)
Sleep quality	
Good	5 (28%)
Average	11 (61%)
Poor	2 (11%)
Very poor	0 (0%)
Do you have dreams	
Yes	8 (44%)
No	10 (56%)
Number of dreams	
One dreams	2 (11%)
Two dreams	2 (11%)
Three dreams	2 (11%)
Forget	2 (11%)
None	10 (56%)
Dream nature	
Not so bad	8 (44%)
No	10 (56%)
Work–related dream content	
Yes	6 (33%)
No	11 (61%)
Forget	1 (6%)
Total sleep duration (hours, mean ± SD)	6.25 ± 1.77

^*^SD, standard deviation.

### Fatigue and mood states

**Figure 1:**
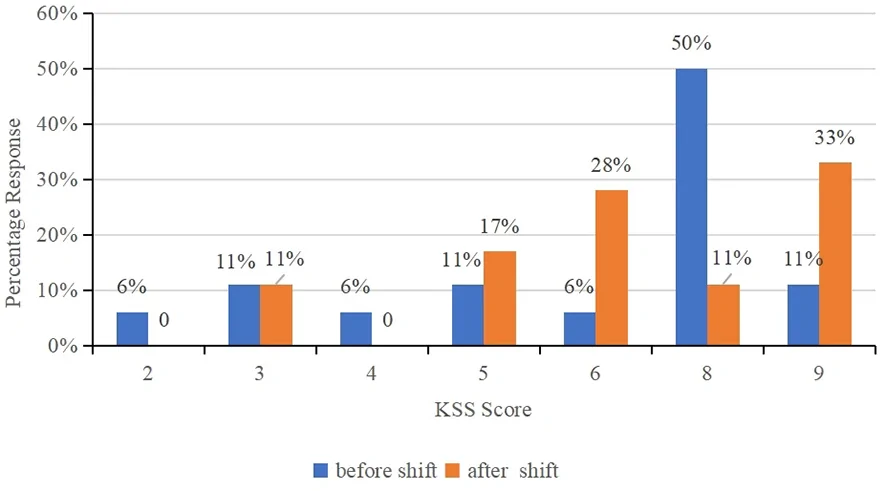
Distribution of Karolinska Sleepiness Scale (KSS) Before and After 24-h Shift. This figure presents the distribution of anesthesiologists' self-reported alertness levels before and after a 24-h on-call shift using the Karolinska Sleepiness Scale (KSS). The KSS levels are defined as follows: 2 = very alert, 3 = alert, 4 = rather alert, 5 = neither alert nor sleepy, 6 = some signs of sleepiness, 8 = sleepy with some effort to stay awake, and 9 = very sleepy with great effort to stay awake, fighting sleep.

As presented in Tables 3, 4, fatigue significantly increased following 24-h duty. POMS demonstrated higher fatigue scores post-shift (*P* < 0.05), whereas other mood-related measures did not show clear significant differences. KSS results (Figure 1) indicated that most participants reported mild sleepiness (scores 2–4) before duty, whereas post-shift scores shifted to higher levels (6–9). Specifically, 50% reported trying to stay awake, 11% were very sleepy but attempting to stay awake, and 33% experienced severe sleepiness with difficulty maintaining alertness. The mean KSS score increased from 6.28 ± 1.96 pre-shift to 7.94 ± 1.47 post-shift (paired *t*-test, *P* = 0.002). Notably, 11% of participants already reported a KSS score of 8 (ut attempting to stay awake, and 33% experienced severepre-existing sleep debt that may have contributed to the elevated post-shift sleepiness levels.

**Table 3:** Big five personality traits before vs. after 24-h shift.

Trait	Before		After		*P*-value
	Mean	SD	Mean	SD	
Adaptation	13.89	2.99	13.94	3.59	0.960
Sociability	15.83	2.18	15.67	2.87	0.846
Openness	12.94	2.53	14.00	3.33	0.291
Altruism	19.00	3.91	19.23	2.91	0.774
Moral sense	19.06	3.02	17.89	2.30	0.201

^*^P < 0.05 indicates statistical significance. SD, standard deviation.

**Table 4:** Mood profile scores before vs. after 24-h shift (POMS).

Mood domain	Before		After		*P*-value
	Mean	SD	Mean	SD	
Tension	4.11	3.20	3.56	5.41	0.710
Angry	2.39	3.38	3.44	5.75	0.507
Fatigue	2.61	3.57	5.89	5.47	0.031^*^
Depression	2.94	4.09	2.94	4.63	1.000
Vigor	9.06	5.48	9.61	4.35	0.738
Confusion	3.44	2.87	3.83	3.90	0.736
Total mood disturbance	101.56	18.32	112.89	32.58	0.207

^*^P < 0.05 indicates statistical significance. SD, standard deviation.

### Sleep duration and physical activity

Wristband monitoring (Table 5) showed an averages of 148.5 min of deep sleep, 119.6 min of light sleep, and 29.0 min of REM sleep. Total sleep was 286.9 min. Physical activity data showed an average of 2,784 steps, 2.12 kilometers walked, and 136.6 kilocalories burned.

**Table 5:** Sleep parameters and physical activity recorded by wristband.

Parameter	Mean	SD
Deep sleep time (min)	148.50	80.11
Light sleep time (min)	119.56	53.04
Rapid eye movement time (min)	29.00	21.21
Total sleep time (min)	286.89	106.92
Step Count	2784.17	2030.53
Distance Walked (km)	2.12	1.60
Calories Burned (kcal)	136.67	101.96

^*^SD, standard deviation.

### On-call workload summary

During 24-h duty, anesthesiologists performed 32 arterial punctures and 37 central venous catheterizations, primarily for catheter replacement, dislodgement, heart failure, cardiac arrest, or myocardial infarction. Emergency intubation was required for 10 patients, with one tracheotomy performed. Analgesia pumps were adjusted for 11 patients, mostly following aortic dissection or cardiac surgery. Procedures occurred in emergency departments, ICUs, and wards. Video laryngoscopes were commonly used. The analgesia regimen consisted of sufentanil 100 μg, dezocine 30 mg, and tropisetron 5 mg in 150 mL saline.

## Discussion

This study confirmed that 24-h on-call duty results in significant sleep deprivation and increased psychological fatigue among anesthesiologists in a specialized cardiovascular hospital. These findings were supported by both self-reported measures and wearable-derived data. Specifically, KSS scores shifted toward greater sleepiness after the duty period, indicating reduced alertness. Similarly, among the POMS domains, fatigue showed the most consistent post-shift change, suggesting that the predominant short-term psychological effect of a 24-h shift may be fatigue rather than a broad deterioration across all mood dimensions. Wearable-derived data also indicated reduced sleep duration and substantial physical activity demands during the monitored duty period. Taken together, these findings highlight the multidimensional burden associated with extended duty hours in anesthesiology training.

In this study, not all measured domains changed significantly after the 24-h duty period. Fatigue showed the clearest difference, whereas other mood-related and personality-related measures did not demonstrate similarly clear changes. This pattern may reflect selective effects of extended duty hours, but it may also be related to limited statistical power given the small sample size and the number of outcomes analyzed. Therefore, the absence of significant differences in some domains should not be interpreted as evidence of no effect.

Fatigue is widely recognized as a risk factor for medical errors and compromised patient safety (9–11). It is defined as a state of tiredness resulting from prolonged physical or mental activity and is closely linked to diminished functional capacity (12). For physicians, particularly those in high-stakes specialties such as anesthesiology, fatigue not only compromises technical performance but also affects emotional regulation, motivation, and communication—core components of clinical care (13–16). Fatigue-induced impairments have been associated with poor decision-making, reduced empathy, and increased rates of absenteeism and burnout (17). Burnout, a more severe form of chronic fatigue, is associated with emotional exhaustion, reduced personal efficacy, and an increased risk of mental health issues (18–21). It also correlates with diminished work ability, defined as the capacity to meet job demands both mentally and physically (22, 23). In acute care and emergency contexts, where clinical judgment and adaptability are vital, even partial sleep deprivation can impair cognitive flexibility, situational awareness, and the capacity to respond rapidly under pressure (24).

Despite numerous studies exploring total sleep deprivation, fewer have focused on the cumulative effects of partial sleep loss, especially in the anesthesiology field. Our study addresses this gap by providing real-world data on the adverse emotional and physiological effects of 24-h shifts. Partial sleep deprivation has also been linked to biological disturbances, such as increased levels of proinflammatory cytokines (e.g., IL-6 and TNF-α) and a significant decrease in cortisol, which may exacerbate cognitive and physical decline (25).

Anesthesiologists must routinely perform complex medical procedures while engaging in effective interdisciplinary communication, often under high-stakes conditions. Night calls with heavy workloads are inherently stressful and may compound personal and professional challenges (24). Therefore, work schedules must be designed to minimize fatigue-induced errors through appropriate recovery periods and mental health support.

These findings support the need for institutional attention to occupational fatigue. Potential strategies may include fatigue monitoring systems, restrictions on consecutive long shifts, mandatory rest periods, and regular assessment of mental wellbeing. Although this study focused on anesthesia fellows, the implications may also be relevant to other high-demand and time-critical medical specialties.

This study has several limitations. First, sleep quality was assessed using a simple self-reported item rather than a validated sleep questionnaire, which may limit comparability with other studies. Second, wearable monitoring was limited to the 24-h on-call duty period and did not include pre-duty or post-duty days; therefore, the study characterizes sleep obtained during the monitored shift rather than habitual sleep patterns or recovery sleep. Third, the wristband used in this study was a consumer device rather than a research-grade actigraphy system, and its sleep-stage estimates should therefore be interpreted cautiously. Finally, the sample size was small relative to the number of psychological and wearable-derived outcomes analyzed. As a result, the study may have been underpowered for some secondary analyses, and the findings should be considered preliminary and confirmed in larger studies.

## Conclusions

This study suggests that anesthesia fellows obtained limited sleep during a monitored 24-h on-call duty period and experienced increased fatigue and sleepiness after duty at a large cardiovascular center. These findings may have implications for physician wellbeing and patient safety and support further investigation in larger studies.

## Data Availability

The original contributions presented in the study are included in the article/supplementary material, further inquiries can be directed to the corresponding author.
