# Correction: The impact of 24-h shift on cardiac anesthesia fellows

**DOI:** 10.3389/fpubh.2026.1988278

**Published:** 2026-09-15

**Authors:** Rongli Liu, Ken Shao, Lixian He, Yuntai Yao

**Affiliations:** 1 Jingmen Central Hospital, Jingmen Central Hospital Affiliated to Jingchu University of Technology, Jingmen, China; 2 Department of Anesthesiology, Fuwai Yunnan Hospital, Chinese Academy of Medical Sciences, Affiliated Cardiovascular Hospital of Kunming Medical University, Kunming, China; 3 Yunnan Provincial Cardiovascular Clinical Medical Center, Kunming, China; 4 Yunnan Provincial Cardiovascular Clinical Medical Research Center, Kunming, China; 5 Department of Anesthesiology, Fuwai Hospital, National Center for Cardiovascular Diseases, Peking Union Medical College and Chinese Academy of Medical Sciences, Beijing, China; 6 Center for Outcomes Research, Department of Anesthesiology, Critical Care and Pain Medicine, University of Texas, Houston, TX, United States; 7 Outcomes Research Consortium, Houston, TX, United States

**Keywords:** anesthesia, anesthesiologist, fellow doctors, on-call shift, questionnaire, sleep quality

Author Ken Shao was erroneously omitted as equal contributing author.

The original version of this article has been updated.

